# The concept of vulnerability in aged care: a systematic review of argument-based ethics literature

**DOI:** 10.1186/s12910-022-00819-3

**Published:** 2022-08-16

**Authors:** Virginia Sanchini, Roberta Sala, Chris Gastmans

**Affiliations:** 1 Department of Oncology and Hemato-Oncology, University of Milan, Milan, Italy; 2 Vita-Salute San Raffaele University, Milan, Italy; 3 Department of Public Health and Primary Care, Centre for Biomedical Ethics and Law, KU Leuven, Leuven, Belgium

**Keywords:** Vulnerability, Frailty, Fragility, Older adults, Clinical ethics, Bioethics, Systematic review

## Abstract

**Background:**

Vulnerability is a key concept in traditional and contemporary bioethics. In the philosophical literature, vulnerability is understood not only to be an ontological condition of humanity, but also to be a consequence of contingent factors. Within bioethics debates, vulnerable populations are defined in relation to compromised capacity to consent, increased susceptibility to harm, and/or exploitation. Although vulnerability has historically been associated with older adults, to date, no comprehensive or systematic work exists on the meaning of their vulnerability. To fill this gap, we analysed the literature on aged care for the meaning, foundations, and uses of vulnerability as an ethical concept.

**Methods:**

Using PRISMA guidelines, we conducted a systematic review of argument-based ethics literature in four major databases: PubMed, Embase®, Web of Science™, and Philosopher’s Index. These covered biomedical, philosophy, bioethical, and anthropological literature. Titles, abstracts, and full texts of identified papers were screened for relevance. The snowball technique and citation tracking were used to identify relevant publications. Data analysis and synthesis followed the preparatory steps of the coding process detailed in the QUAGOL methodology.

**Results:**

Thirty-eight publications met our criteria and were included. Publication dates ranged from 1984 to 2020, with 17 publications appearing between 2015 and 2020. Publications originated from all five major continents, as indicated by the affiliation of the first author. Our analyses revealed that the concept of vulnerability could be distinguished in terms of basic human and situational vulnerability. Six dimensions of older adults’ vulnerability were identified: physical; psychological; relational/interpersonal; moral; sociocultural, political, and economic; and existential/spiritual. This analysis suggested three ways to relate to older adults’ vulnerability: understanding older adults’ vulnerability, taking care of vulnerable older adults, and intervening through socio-political-economic measures.

**Conclusions:**

The way in which vulnerability was conceptualised in the included publications overlaps with distinctions used within contemporary bioethics literature. Dimensions of aged care vulnerability map onto defining features of humans, giving weight to the claim that vulnerability represents an inherent characteristic of humans. Vulnerability is mostly a value-laden concept, endowed with positive and negative connotations. Most publications focused on and promoted aged care, strengthening the idea that care is a defining practice of being human.

**Supplementary Information:**

The online version contains supplementary material available at 10.1186/s12910-022-00819-3.

## Background

The word ‘vulnerability’ derives from the Latin verb vulnerare (i.e., wounding) and from the Latin noun vulnus (i.e., wound). Therefore, etymologically, vulnerability mostly refers to the susceptibility of being physically or emotionally wounded [1, 2]. Yet, besides this prima facie characterisation, the notion of vulnerability has eschewed a univocal definition, both within and across distinct threads of the literature [1, 3–5].

In philosophical literature, vulnerability is mostly considered to be a defining ontological feature of human beings, who are exposed to finitude and subject to the consequences of human embodiment, including the mutual interdependence and/or sociality that inherently structure human life [6–9]. Complementary— or, in some cases, alternative—philosophical approaches have interpreted vulnerability as the consequence of contingent factors, which expose some beings more than others to the aforementioned ‘wounds’, rather than interpreting it as a universal trait of our humanity [10, 11].

In debates within mainstream bioethics, vulnerability is mostly addressed in terms of vulnerable populations, thus shifting the focus from theoretical concepts to groups of individuals. However, defining vulnerable populations or groups has also proved difficult [3], while the concept of vulnerability itself remains ill-defined in such literature. In broad terms, the label ‘vulnerable’ has been attributed to populations characterised by compromised capacity for consent (e.g., patients with serious cognitive impairments); by being at a higher risk of incurring in harm and/or wrongs (e.g., pregnant woman); or by both (e.g., children belonging to certain ethnic groups). Moreover, within research ethics policy and guidelines, vulnerable populations have been defined in relation to ‘reasons for vulnerability’ [12], which can be divided into two broad groups—‘respect for persons-based accounts’ and ‘justice-based accounts’ [12 p. 2]. According to ‘respect for persons-based accounts’, ‘persons who cannot provide informed and voluntary consent are susceptible to harm (i.e., vulnerable), because they are not able to protect their interests’ [12 p. 2]. According to ‘justice-based accounts’, vulnerable populations are those that are potentially more exposed to unfairness during recruitment and distribution of research benefits and risks, as well to exploitation, broadly understood. In addition, overlap between reasons for vulnerability, and hence of categories of vulnerable individuals, may occur. According to these interpretations, vulnerability has a negative connotation, and thus requires some form of intervention, among which ‘extra justification for including vulnerable populations in research’ [13, 14], ‘considered’/‘special’ protection [15, 16], and research and healthcare tailored on vulnerable populations’ needs [15–17] are paramount.

Historically, among the categories of vulnerable people, older persons are predominant. Older adults represent a vulnerable population in public health ethics [18–21], research ethics [16, 17], and clinical ethics domains [22–24]. From a pathophysiological perspective in clinical ethics, an older adult is someone who is in a state of constant cognitive and physical decline [25]. Moreover, this condition leads to progressive depletion of functional reserve mechanisms, reduced homeostatic capabilities, and is frequently accompanied by complex comorbidities, which are often further complicated by geriatric syndromes and frailty [25]. From a psychosocial standpoint, ageing represents a life state characterised by contrasting requests (e.g., protection vis-a-vis autonomy recognition). Moreover, conditions such as income insecurity, lack of access to quality healthcare, unfriendly environments, and psychodynamic factors (e.g., uncertain future and fear of death) may further worsen older adults’ stability and increase their vulnerability. Finally, loneliness, and, in some circumstances, even isolation, may also characterise old age: Many elderly persons live alone, may be widowers, who often live in nursing homes. At the same time, some of their peers remain in their own homes, accompanied by caregivers who become their sole companions [26–29].

Although all these conditions may be thought of as being equivalent to older adults’ vulnerability, no comprehensive or systematic research on vulnerability in older adults has appeared in the literature. As a group, older adults are mostly assumed a priori to be or referred to as being vulnerable in bioethics and research ethics. In some but still limited cases, scholars have proposed different (even incompatible) conceptualisations of vulnerability in the wider bioethics literature that potentially seem to hold relevance for geriatric care. This systematic review provides an overview of these different conceptualisations of vulnerability and discusses their moral relevance, value, and the way that acknowledgments of vulnerability are thought to create obligations of care in the context of geriatric care. The findings of the review will facilitate further philosophical and bioethical research on arriving at a more context-specific and robust conceptualisation of vulnerability in the domain of geriatric care.

## Methodology

To gain a comprehensive overview of the definitions, meanings, and the way that authors use the concept of vulnerability in aged care, we conducted a systematic review of argument-based literature [30, 31]. Argument-based reviews are important for acquiring evidence for better decision-making in the delivery of healthcare, policy development, and conducting medical research [30, 32]. They consist of a curation and evaluation of normative bioethics literature aimed at answering an ethical question.

### Research questions

We formulated the following interrelated groups of research questions:

1. What is the meaning of vulnerability in the ethics literature on aged care?
2. In this context, does vulnerability differ from other similar concepts, such as fragility, frailty, and frailness?
3. What are the ethical arguments and approaches used for discussing vulnerability in aged care ethics?
4. What are the obligations, if any, that follow from the acknowledgment of older adults' vulnerability as reported in the aged care literature?

### Literature search

The four research questions were formulated according to three groups of concepts we devised to organise our literature search (Table 1).

**Table 1:** Groups of organising concepts for searching the literature and their associated database search terms

Group 1: vulnerability	Group 2: target population	Group 3: ethics
Vulnerability; vulnerable; fragility; frailty; frail; frailness	Dementia; aged; aging; ageing; elder; elderly; older people; older adult; old people	Ethics; philosophy; bioethics; anthropology; medical anthropology

These ‘structures’ progressively culled publications to those that allowed us to answer our research question. The purpose of Group 1 concepts was to identify publications that focused on the concept of vulnerability and/or akin concepts, e.g., fragility. The purpose of Group 2 was to help narrow down papers that dealt with the specific population under investigation, i.e., older adults. The purpose of Group 3 was to restrict the search to research domains that likely contained papers using conceptualisations of vulnerability and/or akin concepts. Each group concept was expressed in specific database search terms in a suitable format for the different database queries (Table 2). Research strings were developed by the first author (VS) in consultation with the last author (CG).

**Table 2:** Search strings used for searching databases stratified by organising concepts*

Database	Group 1: vulnerability		Group 2: target population		Group 3: ethics
Pubmed	((((((((((vulnerability[Title/Abstract]) OR vulnerab*[Title/Abstract]) OR fragility[Title/Abstract]) OR frailty[Title/Abstract]) OR frail[Title/Abstract]) OR fragilit*[Title/Abstract]) OR frailness[Title/Abstract]) OR frailties[Title/Abstract])) OR "Frailty"[Mesh]))	AND	((((("Dementia"[Mesh:NoExp] OR dementia [Title/Abstract])) OR ((((((((((aged[Title/Abstract]) OR aging[Title/Abstract]) OR ageing) OR elder*) OR elderly) OR older people) OR older adult*) OR old people)) OR "Aging"[Mesh:NoExp]) OR (("Aged"[Mesh])))))	AND	(((("Ethics"[Mesh] OR "Philosophy"[Mesh] OR ethic* OR philosophy OR bioethic*[Title/Abstract] OR philosophical[Title/Abstract] OR moral[Title/Abstract] OR morals[Title/Abstract]))) OR ("Anthropology, Medical"[Mesh] OR "anthropologic*" [Title/Abstract]))
Embase	vulnerability:ti,ab,kw OR vulnerab*:ti,ab,kw OR fragility:ti,ab,kw OR fragilit*:ti,ab,kw OR frailty:ti,ab,kw OR frailties:ti,ab,kw OR frailness:ti,ab,kw	AND	'aged'/de OR 'aged hospital patient'/de OR 'frail elderly'/exp OR 'institutionalized elderly'/exp OR 'very elderly'/exp OR 'aging'/de OR 'dementia'/de OR aged:ti,ab,kw OR ageing:ti,ab,kw OR elder*:ti,ab,kw OR elderly:ti,ab,kw OR 'older people':ti,ab,kw OR 'old people':ti,ab,kw OR 'older adults':ti,ab,kw OR dementia:ti,ab,kw	AND	'medical anthropology'/exp OR anthropologic* OR 'ethics'/exp OR 'philosophy'/exp OR 'ethics':ab,ti,kw OR 'ethical':ab,ti,kw OR 'philosophy':ab,ti,kw OR 'philosophical':ab,ti,kw OR 'bioethics':ab,ti,kw OR 'bioethical':ab,ti,kw OR 'moral':ab,ti,kw OR 'morals':ab,ti,kw
Web of Science	TS = (vulnerability OR vulnerab* OR fragility OR frailty OR fragilit* OR frailness OR frailties)	AND	TS = (aged OR aging OR ageing OR elder* OR elderly OR older people OR old people OR older adult* OR dementia)	AND	TS = (ethics OR ethical OR philosophy OR philosophical OR bioethics OR bioethical OR moral OR morals OR medical anthropology)
Philosopher’s Index	noft(vulnerability OR vulnerable OR vulnerab* OR fragility OR frailty OR fragilit* OR frailness OR frailties)	AND	noft(aged OR aging OR ageing OR elder* OR elderly OR older people OR old people OR older adult* OR dementia)	AND	noft(ethics OR ethical OR philosophy OR bioethics OR bioethical OR moral OR morals OR medical anthropology)

*See Table 1 for organising concepts

Four major databases were queried: Pubmed®, Embase®, Web of Science™, and Philosopher’s Index. These databases, as a group, cover the literature in biomedicine, bioethics, philosophy, and (medical) anthropology.

Database queries were conducted between the 19th and the 23rd of December 2019, using language filters to identify only articles published in English (Table 3). Table 3 presents the number of results returned using the search terms, as well as the specific days on which the searches were performed.

**Table 3:** Literature search results (‘hits’) for each database and English-only results

Database	Search date	Number of results	Results (English-only filter)
Pubmed	19/12/2019	3758	3498
Embase	23/12/2019	1368	1290
Web of science	21/12/2019	989	898
Philosopher’s index	23/12/2019	51	49
	Total	6166	5735

We used EndNote™ (version X9, Clarivate Analytics, Philadelphia, PA, USA) reference library software to conduct and organise citations of the identified papers; duplicates were manually deleted.

**Fig. 1:**
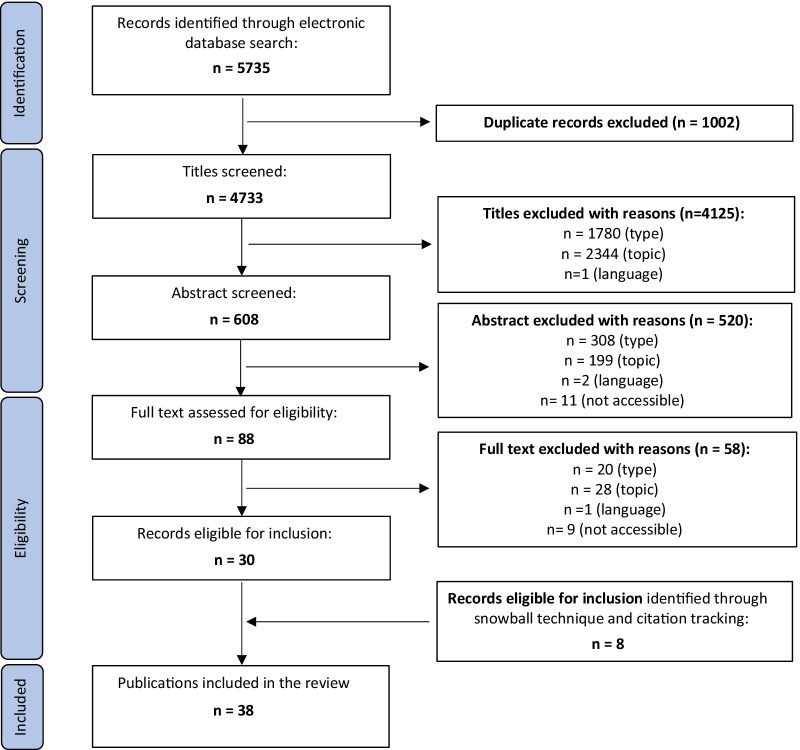
Prisma scheme Flow chart showing electronic database search, publication identification, and selection process for the included articles

The first author (VS) screened the titles, abstracts, and full texts of identified papers, according to a predefined set of inclusion and exclusion criteria (below) [33]. Abstract screening was performed independently by the first (VS) and last (CG) authors to assess the consistency of applying our selection criteria. For 91.94% of the abstracts (559 of 608), we agreed on the items to be included or excluded. For the questionable abstracts (8.06%, corresponding to 49 papers), the first (VS) and last (CG) authors discussed the candidate abstract until agreement was reached. If the full text of an article was not available, we emailed the first or corresponding author of that article to request a PDF copy. We also used the snowball technique and citation tracking to identify additional potentially relevant publications. Our search process was performed according to the statement and flowchart of the Preferred Reporting Items for Systematic Reviews and Meta-Analysis (PRISMA) [33] (Fig. 1).

The final list of included publications is reported in Table 4. The description of characteristics of included publications is reported in Table 5.

**Table 4:** List of included publications and summary descriptions (*N* = 38)

No.*	Author	Country affiliation of first author	Publication year	Professional background of first author	Study focus
38	Agu Francis C	Africa	2013	Bioethics	Vulnerability
39	Baars Jan	The Netherlands	2013	Philosophy	Vulnerability
40	*As above*	*As above*	2017	*As above*	Vulnerability
41	Blasimme Alessandro	Switzerland	2017	Philosophy	Frailty
42	Bozzaro Claudia	Germany	2018	Philosophy	Vulnerability
43	Brocklehurst Hilary	United Kingdom	2008	International relations	Vulnerability
44	Brown Ivan	Canada	1995	Psychology	Frailty
45	Casey Marie S	Australia	1995	Nursing	Vulnerability
46	Denny Dawn L	USA	2012	Nursing	Vulnerability
47	Dunn Michael	United Kingdom	2018	Bioethics	Vulnerability
48	Erlen Judith Ann	USA	2007	Nursing	Vulnerability
49	Gadow Sally	USA	1983	Nursing, Philosophy	Frailty
50	Groenhaut Ruth	USA	2019	Philosophy	Frailty and Fragility
51	Grundy Emily	United Kingdom	2006	Medical Demography	Vulnerability
52	Hardin Sonya R	USA	2015	Nursing	Vulnerability
53	Hoffmaster Barry	Canada	2006	Philosophy	Vulnerability
54	Jecker Nancy S	USA	1991	Philosophy	Vulnerability
55	Jena Yeremias	Indonesia	2014	Philosophy; Bioethics	Vulnerability
56	Kaufman Sharon R	USA	1994	Medical Anthropology	Frailty
57	Körtner Tobias	Austria	2016	Psychology	Vulnerability
58	Laceulle Hanne	The Netherlands	2017	Philosophy	Vulnerability
59	*As above*	*As above*	2018	*As above*	Vulnerability
60	Lloyd-Sherlock Peter	United Kingdom	2006	Geography; Economics	Vulnerability
61	Luna Florencia	Argentina	2014	Philosophy; Bioethics	Vulnerability
62	Makela Petra	United Kingdom	2018	Medical doctor	Frailty
63	Morell Carolyn M	USA	2003	Social Science	Vulnerability and Frailty
64	Niemeyer-Guimaraes Marcio	Brazil	2017	Medical doctor	Vulnerability
65	O’ Brolchain Fiachra	Ireland	2019	Philosophy	Vulnerability
66	Pickard Susan	United Kingdom	2019	Sociology	Frailty
67	Raphael Dennis	USA	1995	Psychology	Frailty
68	Raudonis Barbara M	USA	2010	Nursing	Frailty
69	Rook Karen S	USA	2017	Psychology	Vulnerability
70	Schröder-Butterfill Elisabeth	United Kingdom	2006	Human Sciences; Gerontology	Vulnerability
71	Siegler Mark	USA	1984	Medical doctor	Vulnerability
72	van der Meide Hanneke	The Netherlands	2015	(Cultural) Anthropology; Philosophy	Vulnerability
73	Van Eeuwijk Peter	Switzerland	2006	(Social) Anthropology	Vulnerability
74	Waldon Mandy	United Kingdom	2018	Nursing	Frailty
75	Wareham Christopher Simon	Africa	2015	Bioethics	Vulnerability

^*^Publication identification number

**Table 5:** Detailed characteristics of included publications (*N* = 38)

Analysed features (Number of Publications)/Paper No. as listed in Table 4
1. *Focus*
Vulnerability (27): 38–40, 42, 43, 45–48, 51–55, 57–61, 64, 65, 69–73, 75
Frailty (9): 41, 44, 49, 56, 62, 66–68, 74
(both) Frailty and fragility (1): 50
(both) Vulnerability and frailty (1): 63
2. *Country affiliation of the first author*
Americas (16): Argentina (1): 61; Brazil (1): 64; Canada (2): 44, 53; USA (12): 46, 48, 49, 50, 52, 54, 56, 63, 67, 68, 69, 71
Europe (18): Austria (1): 57; Germany (1): 42; Ireland (1): 65; Switzerland (2): 41, 73; The Netherlands (5): 39, 40, 58, 59, 72; United Kingdom (8): 43, 47, 51, 60, 62, 66, 70, 74
Africa (2): 38, 75
Australia (1): 45
Indonesia (1): 55
3. *Year of publication*
2020–2015 (17): 40–42, 47, 50, 52, 57–59, 62, 64–66, 69, 72, 74, 75
2014–2010 (6): 38, 39, 46, 55, 61, 68
2009–2005 (7): 43, 48, 51, 53, 60, 70, 73
2004–2000 (1): 63
Before 2000 (7): 44, 45, 49, 54, 56, 67, 71
4. *Population under investigation*
Older adults, no specified age and/or gender (22): 39, 40, 42–45, 47, 49–51, 53, 56–59, 66–71, 74, 75
Older adults, specified age (4): 38 & 41 (+ 65-years-old), 46 (70 and above), 48 (80 and above)
Older adults in ‘upper middle’ income countries (according to the country list, World Bank) (5): 55, 70, 73 (Indonesia), 60 (Thailand); 61 (Brazil & Argentina)
Female older adults (2): 54, 63
Hospitalised older adults (2): 52, 72
Older adults with dementia (1): 65
Residents of nursing homes (1): 62
Cancer, older patients (1): 64
5. Main ethical approach* (21)
Phenomenology and existentialism (5): 42, 45, 49, 66, 72
Care Ethics (4): 48, 50, 56, 72
Feminist Ethics (4): 50, 54, 62, 63
Principlism (3): 38, 46, 57
Capability Approach (2): 41, 47
Public Health Ethics (2): 61, 67
Virtue Ethics (2): 58, 65
Analytic political philosophy (1): 75
Personalistic Ethics (1): 55
Articles that do not explicitly refer to a specific ethical theory or approach (17): 39, 40, 43, 44, 51–53, 59, 60, 64, 68–71, 73, 74

*A single article can be represented more than once

### Inclusion and exclusion criteria

To be included in our systematic review and appraisal, candidate publications had to meet all inclusion criteria and had to have no exclusionary ones (Table 6). Screening of the publications was not limited by publication date.

**Table 6:** Inclusion and exclusion criteria for selection of the articles

	Criteria	
	Inclusion	Exclusion
Topic	Significant use of the concept of vulnerability and/or related concepts (i.e., frailty, frailness, fragility)	Use of the concept of vulnerability and/or akin concepts was not significant, and/or it was used in a purely medical sense
Research domain	Application to the specific field of aged care (broadly understood)	Reference to fields other than aged care (e.g., research ethics)
Population	Reference to older adults	Target population is not represented by older adults
Language	Publication must be in English	Publications in languages other than English
Types	Publication is considered to be in the argument-based literature; i.e., an article using ethical concepts derived from current or traditional ethical theories in order to argue for a position or conclusion	Editorials, book chapters, position papers, guidelines, reviews, protocols, ethics policies, and ethics codes

As there is no standard for quality appraisal of papers in the argument-based literature, we referred to the ‘appraisal using procedural quality assurance criteria’s strategy’ of Mertz [31]. Quality appraisal was not used as a tool for inclusion/exclusion. Since the articles were published in peer-reviewed journals, we relied on the standards of the peer review process and on the academic reputation of the journals to bolster our assumptions that the quality of selected publications was sufficiently good.

### Data extraction and synthesis

Data analysis and synthesis adhered to the five preparatory steps of the coding process detailed in the Qualitative Analysis Guide of Leuven (QUAGOL) [34, 35]. QUAGOL has been used in other argument-based reviews [32, 36, 37].

For data extraction, the first author (VS) first read and reread several times all the included publications, highlighting the relevant parts and the main arguments described. The same author then developed a narrative summary of these highlighted parts. Third, for each publication, a conceptual scheme was created. The conceptual scheme is a synthetic framework in which different concepts deemed relevant to answer the research questions are presented [34, 35]. An example of a conceptual scheme is presented in Additional file 1: “Example of conceptual scheme”. Each conceptual scheme was analysed separately by the first (VS) and last (CG) authors to verify that it accurately characterised each included publication. Both researchers discussed conceptual schemes until they agreed on their adequate content. Fourth, these individual conceptual schemes were compared, with the aim of uncovering relationships that would produce an overall answer to our research questions. This resulted in a comprehensive scheme that integrated the most relevant meanings and dimensions of vulnerability and related concepts in aged care ethics (Tables 7 and 8). This scheme was iteratively evaluated and checked against previous QUAGOL steps to ensure that it adequately reflected the included papers. Finally, we synthesised a description of these results.

**Table 7:** Global scheme emerging from analysis of the 38 included publications (part one)*

	Included articles
*1. Conceptualisation of vulnerability (and related concepts), generally understood*	
1.1 Basic human vulnerability (*N* = 12) Vulnerability (and related concepts) as an intrinsic trait of human nature	38–40, 42, 49, 50, 53, 55, 58, 59, 64, 65
1.2 Situational vulnerability (*N* = 8) Vulnerability (and related concepts) as a consequence of extrinsic cultural, social, political, economic factors	41, 44, 51, 52, 55, 61, 66, 68
1.3 NO conceptualisation of vulnerability, generally understood (*N* = 18)	43, 45–48, 54, 56, 57, 60, 62, 63, 67, 69–75
*2. Conceptualisation of older adults’ vulnerability (and related concepts)*	
2.1 Aging as a condition of increased basic human vulnerability (*N* = 9)	38–40, 44, 49, 53, 58, 59, 65
2.2 Aging as a condition of increased situational vulnerability (*N* = 25)	41–43, 46, 47, 48, 51, 52, 54, 56, 57, 60–63, 66–74
2.3 Aging as a condition of both increased basic human & situational vulnerability (*N* = 4)	45, 50, 55, 64
*3. Dimensions of aged care vulnerability (and related concepts)*	
3.1 Physical Vulnerability (*N* = 14)	
Non pathological physical/physiological bodily deterioration related to ageing	43, 44, 48, 50, 52, 53, 54, 61, 62, 67, 68, 70, 74
Pathological physical/physiological conditions related to ageing	43, 50, 52, 61, 65
3.2 Psychological Vulnerability (*N* = 18)	
Strictly psychological	Cognitive 43, 44, 48, 50, 52, 61, 65, 67, 68
	Emotional 40, 48, 54, 57, 61, 69, 72
	Personal traits 70
Experiential	42, 53, 63, 72
3.3 Relational/interpersonal vulnerability (*N* = 14)	
Ontological interdependence of the human condition	40, 42, 58, 73
(Inter)dependence in real-world settings	43, 44, 51, 56, 57, 61, 65, 67, 70, 74
3.4 Moral vulnerability (*N* = 7)	
Positive view	58, 59, 65, 68
Negative view	61, 62, 64
3.5 Socio-cultural, political, and economic vulnerability (*N* = 22)	
Socio-cultural	Broadly understood 41, 50, 51, 55, 68, 70, 73
	Isolation/exclusion from social life 42, 43, 45, 62, 65
	Marginalisation (also related to racism) 43, 48
	Stigmatisation (i.e., ageism) 43, 48, 52
	Related to gender 54, 73, 74
	Low education level attained 70, 74
Political and economic	43, 44, 47, 50, 51, 54, 55, 60, 61, 71, 73, 74, 75
3.6 Existential/spiritual vulnerability (*N* = 15)	
Trait of human existence	44, 49, 53, 55, 66
Experience of human finitude	38, 42, 55, 58, 66
Acquisition of new meaning(s)	40, 58, 59, 63, 68
Loss of meaning	43, 45, 67, 68
*4. Additional conditions/issues correlated to aged care vulnerability (and related concepts)*	
4.1 Depression	43, 44, 68
4.2 Loneliness	45, 54, 66
4.3 Undertreatment of pain	46, 68
.4.4 Medicalisation	56, 62
4.5 Living Situation	44, 52, 61, 62, 67, 70, 72, 74
4.6 Control	56, 65

^*^A single article can be represented in more than one category

**Table 8:** Global scheme emerging from analysis of the 38 included articles (part two)*

Means to address vulnerability				
Understanding older adults’ vulnerability (i)	Taking care of vulnerable older adults (ii)	Intervening through socio-cultural, political, and economic measures (iii)	Both (i) and (ii)	Both (i) and (iii)
*Ethical approach †*				
Phenomenology and existentialism (42, 45, 49, 66, 72)	Principlism (38, 46, 57)	Capability approach (41, 47)	Care ethics (48, 50, 56, 72)	Personalistic ethics (55)
		Feminist ethics (50, 54, 62, 63)		
Virtue ethics (58, 65)		Public health ethics (61, 67)		
		Analytic political philosophy (75)		

*A single article can be represented in more than one category

^†^publication No. as listed in Table 4

## Results

### General description of publications

Thirty-eight publications met our inclusion criteria (identified by Nos. 38-75 in Table 4). For a detailed description of the general characteristics of the included publications, see Table 5. All the included publications belong to argument-based literature; thus, they provide conceptual insights on vulnerability and related concepts (Table 6). With respect to the latter, the majority of papers (*N* = 27) presented vulnerability as its object of investigation [38–64]; nine publications referred to frailty [65–73]; while two publications focused on both frailty and fragility [74], and vulnerability and frailty, respectively [75]. Although these are different terms, we considered it acceptable to include publications focusing not only on vulnerability but also on these other two notions. For these to be included, two conditions had to be met: (i) frailty and fragility were used synonymously with vulnerability; and (ii) they were used within argument-based reasoning and not strictly as medical notions or description.

Publication dates ranged from 1984 to 2020, with 17 articles published between 2015 and 2020 [40, 41, 45, 48, 52–54, 57–59, 62, 64, 65, 69, 70, 73, 74]. Studies from the Americas, Europe, Australia-Oceania, Asia, and Africa are represented in this systematic review; that is, the first author and/or corresponding author was from one of these continents. However, the vast majority of papers came from studies conducted in Europe (*N* = 18) and the Americas (*N* = 16). Even within Europe and the Americas, the authors’ affiliations varied widely. For authors based in Europe, the authors’ affiliation for 13 of the included publications was from an institution in Britain [42, 45, 47, 55, 60, 69, 70, 73] or The Netherlands [39, 40, 53, 54, 62]. For authors based in the Americas, the affiliation for 12 of 16 of the included publications was from an institution in the US [44, 46, 48, 50, 59, 61, 67, 68, 71, 72, 74, 75].

To be included in this review, papers had to have referred to older adults in aged care, that being a broadly understood term. Most papers (*N* = 22) referred to the category ‘older adults’, without specifying their age, gender or, above all, whether they are affected by a medical condition. Five papers were derived from studies that investigated the condition of older adults living in upper-middle income countries [51, 55, 56, 60, 63].

While doing data extraction and synthesis, we realised that most authors of the included publications argued from a specific ethical stance in their paper. We identified nine ethical approaches that authors used. The arguments used in two papers were informed by several approaches, and thus their publications were categorised into more than one ethical approach [62, 74]. We also identified several ‘outlier’ publications. These are papers that could not be readily categorised into any one of the nine ethical approaches. However, these outliers still clearly dealt with ethical arguments concerning the topic of vulnerability in aged care.

Our analysis and synthesis supported a fivefold structure of the included publications (Tables 7 and 8). This structure could be conceived of as sections. The first section outlines how the concept of broadly defined vulnerability was addressed. The second section focuses on vulnerability in aged care, thus showing how the different accounts of broadly defined vulnerability find their counterpart in this narrower debate. Drawing from the adjusted understanding of the first two sections, the third and fourth sections are manifested as a theoretical conceptualisation of vulnerability in aged care, explaining in detail its defining dimensions. The fifth section relates the findings concerning the means to address aged care vulnerability, with the ethical approaches adopted in the same publications. This showed the relationships between ethical approaches and corresponding strategies for addressing vulnerability in aged care.

### Conceptualisation of vulnerability and related concepts: how they are generally understood

The majority of papers (*N* = 20) engaged in the well-known debate over the definition and conceptualisation of vulnerability, as it is broadly understood. This debate encompassed the question about what the concept of vulnerability refers to, in particular, whether it refers to a condition generally affecting human beings as such (namely, what we referred to as ‘basic human vulnerability’) or whether there are some specific situational conditions that make some human beings more vulnerable than others (namely, what we referred to as ‘situational vulnerability’) (Table 7).

In 12 out of the 20 papers, vulnerability was defined in terms of basic human vulnerability. Accordingly, most authors considered vulnerability to be a normative concept related to the very essence of our anthropological condition [41] and a necessary attribution intrinsically affecting humankind [38, 39, 67, 74]; they conceived of this in different ways [67, 74]. Thus, for this definition, being vulnerable means first and foremost being exposed to the experience of ‘human finitude’ [38, 39, 53, 54], which in turn is related to the certainty of death and the ‘fundamental uncertainty’ characterising the human condition [40]. Basic human vulnerability means also being open to the possibility of being affected in life by both pleasures and sufferings, as well as experiencing the condition of inter-human dependency [40]. This latter is also related to the progressive loss of power and/or control that we, as humans, experience during life [49]. Despite being an innate human trait, basic human vulnerability does not equally characterise all human beings in the same manner, but it varies depending on the individual subject and/or life conditions a subject is experiencing [58, 67, 74]. In this sense, human vulnerability can change over time and in the presence of potential stressors related to poor functional decline; this means that it can be repaired or can worsen [38].

The authors of the remaining eight papers argued for a definition of vulnerability that is in line with what we term situational vulnerability. According to this definition, vulnerability does not refer to a trait characterising human beings as such, but it describes the condition affecting only some agents who are more likely to be harmed and/or injured than others [47, 65, 66, 72], due to situational contingent circumstances; these can be social, political, or economic [51, 56]. This notion of vulnerability has also a normative connotation, endowed with negative characterisation related to a condition of minority [72].

The remaining 18 papers were not relevant for this debate about broadly defined vulnerability; they focused only on the concept of vulnerability in the context of aged care.

### Conceptualisation of older adults’ vulnerability

All the publications included in this systematic review conceptualised vulnerability in the context of aged care. We noticed that older adults’ vulnerability was presented in terms of one of the three possible conditions: (i) increased basic human vulnerability, (ii) increased situational vulnerability, (iii) both increased basic human *and* situational vulnerability.

Authors discussing ageing in terms of condition (i) (*N* = 9) argued that traits representing humans as vulnerable—that is, experiencing finitude, being subject to progressive deterioration, and mutual inter-dependence—are amplified when people become old. Accordingly, our analyses showed that being old primarily means experiencing more intensely the state of human finitude because of a heightened and closer ‘connection with our own mortality’ [39, 40, 66]. Being old also means being subjected to a progressive loss of independence, which appears as being totally compromised in some medical conditions (e.g., dementia); this, in turn, progresses to a greater mutual interdependence [58].

However, the majority of publications (*N* = 25) considered older adults’ vulnerability in terms of condition (ii); that is, vulnerability is the consequence of one or more situational conditions that may accompany old age. These conditions make older adults more ‘exposed’ than other categories of agents to threats or injustices. There may be different kinds of situational conditions, spanning from medical to social, from political to economic conditions, and these more likely result because of their overlap [45, 50, 55, 63]. In line with this characterisation, older adults may be defined as vulnerable, for example, when they are subjected to ageism; that is, they are discriminated against in many contexts, including healthcare provision, simply due to their chronological age [61, 64].

Finally, a small number of publications (*N* = 4) defined older adults’ vulnerability in terms of two dimensions, which is the result of both basic human and situational circumstances [43, 51, 57, 74]. This two-dimensional vulnerability is based on the idea that there are some situations and practices that add more layers of contingent vulnerability to the aged condition [57]. This layering is in addition to the vulnerability intrinsic to the human condition.

### Dimensions of aged care vulnerability

By analysing in detail both basic human and situational conditions that make older adults vulnerable, we identified in the included papers six dimensions of older adults’ vulnerability: (i) physical; (ii) psychological; (iii) relational/interpersonal; (iv) moral; (v) sociocultural, political, and economic; (vi) existential/spiritual vulnerability.

A first intuitive dimension of aged care vulnerability is represented in the notion of physical vulnerability (*N* = 14 publications). It is physical in the sense that bodily deterioration (i.e., non-pathological and pathological physical/physiological decline) tends to disproportionately affect older adults. Non-pathological age-related deterioration are conditions that can potentially lead to the fragility syndrome: physical instability, reduced mobility, loss of power, problems with vision and hearing, among others. Pathological deterioration, on the other hand, refers to specific pathological conditions that may only affect some specific older individuals, such as illness [56], dementia [42, 58, 74], and disability [48].

A second dimension of aged care vulnerability is psychological vulnerability (*N* = 18 publications). Here, we identified both strictly psychological and experiential components, the latter only if correlated with and/or impacting the psychological dimension of humans. Older adults appear psychologically vulnerable because they are subjected to mental health changes [42] and to progressively diminishing intellectual functioning [66]. In addition to these cognitive factors, emotional factors also add further layers of vulnerability [40, 46, 50, 52, 56, 59, 62]. For example, the cumulative loss of loved ones [40], the absence of emotional support towards the end of life [50], and the presence of negative social ties [47] are only some of the conditions that lead older adults to perceive themselves as being psychologically vulnerable. The experiential component deals with the concept of embodiment [41, 49, 62, 75] and with the psychological implications resulting from a shift from ‘an absent to a non-absent body’ [41], which predominantly occurs in old age. This body no longer supports the older one in his/her daily activities; rather, it becomes a matter of concern and requires assistance and care. Older adults also feel psychologically vulnerable because their ‘decaying body’ impacts their personal representation [75].

A third dimension of aged care vulnerability is termed relational/interpersonal vulnerability (*N* = 14 publications). This category deals with the impact of human interdependence in late adulthood and how that relates to vulnerability. Our analysis showed that this concept could be interpreted in two ways: first, as ontological interdependence; second, as interdependence/dependence in real-world settings. While the first interpretation applies to humans in general (and by extension to older adults), the second focuses on the ageing population specifically. Ontological interdependence means recognising the intrinsic relational and social nature of human beings [41]. Such recognition has two main implications. First, being social creatures, individualistic behaviours are against our nature [40]. Second, because we are intrinsically relational—i.e., as humans, we cannot easily avoid relations with other humans—we are also vulnerable. This latter vulnerability is considered in our findings both as a positive feature (e.g., we perceive ourselves as sharing the same condition as vulnerable beings) [53, 63] and a negative feature (e.g., we all suffer the cumulative loss of loved ones) [40, 41].

However, most publications focus on relational/interpersonal vulnerability in terms of interdependence/dependence in real-world settings. Older adults are vulnerable insofar as they progressively become ever more dependent on others. Indeed, due to progressive decline in their physical and cognitive abilities, older adults lose more and more control over their own daily activities [71], and, in broad terms, lose their decision-making capacity [52, 68]. In addition to this vulnerability characterising all older adults, there are some specific conditions that make some older adults more susceptible to further relational/interpersonal vulnerability. This may occur in the case of dependent older adults lacking adequate family support [47, 60, 71] and/or in the case when they have to live alone [73].

A final aspect of relational/interpersonal vulnerability is found in the sexuality of older adults. As some findings suggest, there is a stigma attached to the expression of sexuality in older adults because of the pervasive belief that older adults are not sexually active or that they are necessarily in heterosexual relationships [42, 58].

A fourth dimension of aged care vulnerability is what we defined as moral vulnerability (*N* = 7 publications). Moral vulnerability can take on two meanings. In its positive sense, moral vulnerability is interpreted as care for older adults’ dignity [58], respect for their acquired wisdom [53, 54], and attention to their moral preferences [72]. In its negative sense, it points to the risk for older adults of being subjected to infantilisation. Infantilisation occurs when empathy goes unexpressed and when older adults are treated as objects [56]. Infantilisation also underlies the phenomenon of depersonalisation; that is, when older adults are deprived of their personal identity [69]. Our analysis also revealed that moral vulnerability in its negative sense underlies the stigmatisation of some practices that may help this population to better cope with their personal conditions, such as preventing older adults from receiving palliative care, causing them unnecessary pain [57].

Most publications considered older adults’ vulnerability to emerge as a result of unfair or unjust sociocultural, political, and economic conditions (*N* = 22 publications). Considering only the sociocultural dimension of this vulnerability, older adults become vulnerable when they are excluded from social life and/or are isolated [41–43, 58, 69]; marginalised [42, 46]; and stigmatised [42, 46, 48]. Sociocultural vulnerability affects more females than males [50, 63, 73], and people with low levels of education compared to those who have achieve high levels of education [60, 73]. Low levels of social support are also frequently mentioned as a condition for sociocultural vulnerability [47, 51, 65, 74]. Strictly related to sociocultural dimensions, some publications explicitly referred to forms of economic and political vulnerability [42, 45, 47, 50, 51, 56, 61, 63, 64, 66, 73, 74], the latter including also discrimination in the provision of healthcare [61, 63, 64, 73], and subjection to unjust judicial systems [56].

The last dimension of older adults’ vulnerability we identified is existential/spiritual vulnerability (*N* = 15 publications). This kind of vulnerability is part of the broader category of basic human vulnerability. It refers to those existential and/or spiritual conditions that quintessentially characterise humans but are even more represented in the ageing population [49, 51, 66, 67, 70]. Older adults are existentially and spiritually vulnerable, because they often more vividly experience human finitude [38, 41, 51, 53, 70]; that is, they feel closer to dying and they also disproportionately experience disruption of everyday competences [70]. Their vulnerability thus appears as a lens through which human experiences are intensified [67 p. 144]. Older adults’ existential/spiritual vulnerability is also intrinsically related to the concept of ‘meaning’. In its positive sense, older adults’ vulnerability is characterised in a way that allows life experiences to be interpreted as meaningful [40, 53, 54, 72, 75]. In line with this first interpretation, being old represents an opportunity for human authenticity [54]. In other publications the dimension of existential/spiritual vulnerability may have a negative connotation. In this case, older adults experience meaninglessness and hopelessness [42, 43, 71, 72].

### Additional conditions/issues correlated with aged care vulnerability

In addition to the six dimensions of older adults’ vulnerability, we found that authors indicated that there are also some conditions correlated with older adults’ vulnerability. These may belong to different dimensions simultaneously. These other conditions are depression, loneliness, undertreatment of pain, medicalisation, living situation, and control.

Depression relates to both physical/physiological [42] and psychological vulnerability [53, 66] in terms of its interpretation as a medical or psychological condition.

Loneliness is considered to be one of the main factors correlated with vulnerability. As related to psychological/experiential vulnerability, it appears as the condition that enables older adults to confront the feeling of their own nothingness and/or uselessness [43]. As related to relational/interpersonal vulnerability, it refers to the reality that most older adults are often alone and deprived of satisfying connections with others [43]. As related to existential/spiritual vulnerability, it recalls the ontological separation between human beings [70].

Undertreatment of pain and medicalisation are interesting concepts, since they are not only related to physical vulnerability [44, 72] and psychological/experiential vulnerability [68], respectively, but also can be interpreted as socioculturally sensitive phenomena. This means denying an older person pain relief is considered by some to be a virtuous act [44], while vulnerability understood as a medical condition confers ‘the legitimation that others have to intervene in older adults’ life [69 p. 1044].

When authors refer to living situation, they mean those conditions, either environmental or social, that may create vulnerable situations for older adults. This applies not only to older adults living in dangerous areas and/or unsafe environments [48, 56, 60, 66, 71, 73], but also to those who are disempowered because being subjected to unnecessary hospitalisation [56, 62, 69], for example.

A final factor of vulnerability considers the condition of control. Becoming old leads to the progressive (and physiological) loss of power and control [49, 66]. However, besides this condition, some older adults considered themselves illegitimately surveilled by others [58, 68].

### Means to address aged care vulnerability

Our analysis revealed a close connection between the ethical perspective endorsed by the authors and the suggested approach to address a given vulnerability (Table 8). In other words, the ethical perspective through which vulnerability is conceptualised informs the suggested ways to account for or address vulnerability itself.

Our analysis identified three ways, or strategies, to relate to older adults’ vulnerability: (i) understanding older adults’ vulnerability, (ii) taking care of vulnerable older adults, (iii) intervening through sociopolitical and economic measures.

Putting aside the ‘outlier’ publications (see “General description of publications” section), strategy (i) is advanced by authors who endorse phenomenology and existentialism, and virtue ethics perspectives. Strategy (ii) is advanced by authors who endorse a principlist approach. Strategy (iii) is advanced by authors who endorse a capability approach, feminist ethics, public health ethics, and analytic political philosophy. Finally, mixed strategies were also evident: Supporters of care ethics hold that older adults’ vulnerability requires both (i) and (ii) strategies, while supporters of personalistic ethics hold that older adults’ vulnerability requires both (i) and (iii).

### Understanding older adults’ vulnerability

Proponents of phenomenology and existentialism, and virtue ethics claim that the best way to address older adults’ vulnerability is to first understand it. In five publications [41, 43, 46, 62, 67], the authors endorsed an approach that combines aspects of phenomenology and existentialism. Sensitivities of phenomenology and existentialism consider as a grounding assumption the presence of an inevitable layer of vulnerability, namely basic human vulnerability. At the same time, they argue that additional (situational) layers of vulnerability can emerge only within the first-person perspective of the older adult and his/her experiences. While vulnerability as a metaphysical status characterises all human beings and may be generalised [43], vulnerability as a situational condition appears as a unique and non-generalisable tenet of the specific older adult under consideration [41, 43]. Addressing older adults’ vulnerability therefore requires understanding the very essence of older adulthood as being intrinsically affected by some layers of basic human vulnerability [67, 70]. However, it also means addressing it by thoroughly investigating the inner feelings and sensation of specific older adults, all of whom likely have heterogeneous experiences [41, 62].

Two publications [53, 58] ground their claims in a virtue ethics approach, the latter interpreted using elements of accounts of Aristotle, MacIntyre, and Swanton. Within a virtue ethics approach, vulnerability as an existential condition has a very important role. Indeed, given that vulnerability, dependence, and affliction are intrinsic human characteristics, the underlying philosophical assumption of virtue ethics is that it is essential to recognise and experience existential vulnerability in order to fully express one’s human nature. However, it is precisely the exercise of virtues that enables individuals ‘to confront and respond to vulnerability and dependence in ourselves and others’ [58 p. 968]. Virtues and vulnerability are therefore strongly correlated. According to a virtue ethics approach, older adults’ vulnerability should be understood as an existential condition, not necessarily having a negative connotation. Moreover, since it emphasises life-encompassing moral development, virtue ethics assumes the potential for development to unfold throughout the entire life course. This means taking older individuals seriously as moral agents [53].

### Taking care of vulnerable older adults

Three publications [38, 44, 52] used a principlist approach to ethics. Principlism represents the major approach of contemporary Anglo-Saxon biomedical ethics. Functioning ‘as an analytic framework of general norms derived from the common morality that form a suitable starting point for biomedical ethics’ [76 p. 13], principlism is based on four prima facie moral principles, namely respect for autonomy, non-maleficence, beneficence, and justice. Respect for autonomy is clearly linked to humans’ self-determination, which is usually defined as allowing one to make informed decisions about one’s own life and permitting one to act accordingly. Non-maleficence is essentially a translation of the Hippocratic oath, primum non nocere (i.e., first of all do not harm), while beneficence refers to the consequentialist duty of maximising the patient’s benefit. The principle of justice regulates the relationships between individuals regarding health, requiring also to apply fair procedures in the allocation of health resources across individuals.

Our analysis showed that authors adopting principlism argued that the best means to address older adults’ vulnerability is to protect vulnerable individuals [38]. Protection is intrinsically related to respect for persons. As the Belmont Report suggests [13], individuals incapable of self-determination should be granted appropriate protection. Being incapable of self-determination is one of the conditions defining vulnerability. We also observed that protecting older adults means preserving older adults’ capacity of self-determination (i.e., autonomy) as much as possible, while also promoting their well-being [52]. One threat to well-being in older adulthood is untreated pain. Protecting vulnerable adults also means actively promoting older adults’ pain relief [43].

### Intervening through sociopolitical and economic measures

A third proposed strategy to address older adults’ vulnerability is to intervene through sociopolitical and economic measures so as to minimise vulnerability as much as possible. Supporters of this view advocate four approaches: the capability approach, feminist ethics, public health ethics, and analytic political philosophy. Precisely because they believe that older adults’ vulnerability is the result of unfair sociopolitical and economic contingent conditions, these authors endorse a situational definition of vulnerability. It follows, then, that vulnerability may be reduced by intervening through these approaches.

Two publications endorsed a capability approach [45, 65]. This normative framework discerns three main elements—well-being, functioning, and capabilities—and argues that freedom to achieve well-being is of primary moral importance. Well-being is interpreted in terms of people’s capabilities and function. Looking at the debate over aged care vulnerability through the lens of the capability approach means first to individuate older adults’ well-being, functioning, and capabilities. Our analysis showed that older adults’ well-being corresponds to autonomy, broadly considered. Functioning means remaining autonomous, while capabilities refer to the set of social assets that prevent older adults from losing their autonomy [45, 65]. According to the capability approach, older adults’ vulnerability is neither a descriptive (and necessary) condition correlated to ageing, nor is an existential phenomenon. Vulnerability, however, is a normative status that public health authorities must properly address and, if possible, prevent [65].

A similar answer comes from the proponents of public health ethics approaches [56, 71] and analytic political philosophy [64]. These approaches are characterised by the conviction that health is not only an individual condition, resulting from the biological constitution of the individual herself, but is a complex phenomenon that results additionally from unjust economic systems. When applied to middle income countries [56], public health ethics is narrowly defined as the domain of ethics investigation interested in ‘social justice, poverty, and systematic disadvantage’ [56 p. 186]. Within these approaches, vulnerability is not only a situational but also a ‘multi-layered condition’ [56], that is, the results of different simultaneous conditions. This means that vulnerability should be disassembled to reveal its constituent components in order to find contextual and targeted strategies to address them (e.g., against economic vulnerability, improving the pension system; against housing vulnerability, increase investments in geriatric institutions). It also means that a ‘one-size-fits-all’ solution is inappropriate to address older adults’ vulnerability, the latter being the result of contingent and situational conditions. In other words, vulnerability ‘is seen as a condition of lived experience reflecting the intersection of unique individual factors and proximal and distal environmental factors’ [71 p. 225]. The publication informed by an analytic political philosophy approach [64] focuses on discrimination in health and argues that such discrimination, often labelled as ageism, may be the most important cause of older adults’ vulnerability, even in high income countries.

Feminist ethics approaches [50, 69, 74, 75] apply the entire body of reasoning typical of public health ethics and analytic political philosophy approaches to the debate over gender. They stress that older woman are the ones mostly disadvantaged, the most vulnerable among the vulnerable. This is because  more older women are materially poorer than men. Because women have a higher life expectancy, they spend part of their life without a spouse. Because women live longer than men, they present more comorbidities than men. Finally, they are usually subjected to a hostile cultural climate (e.g., are mostly considered on the basis of their physical characteristics, youth, sexual and/or reproductive functions). Against such a backdrop of vulnerabilities, authors advocating these approaches suggest that intervention should be done through sociopolitical actions. On the one hand, they promote equal treatment, equal opportunity, and equal respect at the public level to reduce existing gender-based inequalities. On the other hand, they work on those ‘social interrelations and organizational practices, through which agency is dispersed’ [69 p. 1045].

In addition to these situational vulnerabilities, one publication [75] also points to the impact that a ‘decaying body’ has for older women. A ‘decaying body’ appears to be more difficult to accept, because of its role in relational and care practices, as well as in reproductive practices. That author proposes to recover what she labels as an ‘empowered feminist ethics’. This effort should not only help older women to ‘deal with the bodily changes that are authentically connected to aging’ [69 p. 79], but also to intervene on a public level. The latter would aim to promote actions that change the social environment in which such convictions originate [69, 75].

### Mixed answers to older adults’ vulnerability

Our analysis showed that authors using care ethics and personalistic ethics approaches proposed addressing older adults’ vulnerability by employing mixed strategies. Care ethics is an approach grounded on the conviction that there is moral significance in the fundamental elements of (care) relationships and dependencies in human life. Accordingly, care ethics seeks to maintain and promote care relationships by contextualising the well-being of caregivers and care-receivers in a network of social relations. Proponents of the care ethics perspective argue that vulnerability must be properly understood and ‘digested’. At the same time, it remains extremely important to protect vulnerable older adults, balancing instances of protection with the older adults’ desire of autonomy [46]. It is precisely within care relationships, and through an empathic understanding between caregivers and care-receivers [46], that older adults’ vulnerability may be properly understood [74]. Such vulnerability has a twofold nature, one endowed with both situational and intrinsic elements. For situational elements, it is important to realise that vulnerability may become a social construction, one emerging during the healthcare encounter [68]. Indeed, ‘the transformation from lived problem to diagnosis, then to treatment plan, then to rules about what ought to be done, and finally to negotiated compliance is the form the social construction of frailty takes in the context of health care’ [68 p. 54]. However, older adults, especially when hospitalised, are also intrinsically vulnerable. This is because their vulnerability results from complex lifeworld dimensions, including mood (i.e., their inner state); embodiment (i.e., their physical as well as their lived body dimensions); intersubjectivity (i.e., how they are in relation with others); and space and time (both objectively and subjectively interpreted) [62].

Finally, one publication [51] was informed by a personalistic ethics approach. Personalistic ethics draws from Schotsmans’ account (1999) and states that persons are unique and original, relational and inter-subjective, and that communication and solidarity are very important for human beings [51]. The author endorsing the personalistic ethics approach argued that vulnerability as an existential condition should be understood in its complexity. However, when situational elements are also present—namely, lack of economic security, health problems, insufficient social support—these should be actively addressed through focused sociopolitical and economic actions. The situational elements affect some older adults, in particular, those in developing countries.

## Discussion

Although concerns for vulnerability are central to bioethics reflection, the concept of vulnerability is still mostly under-theorized in the corresponding literature [4], especially in the context of aged care. We addressed this theoretical shortfall in the present systematic review of argument-based ethics literature by answering four research questions about the meaning of vulnerability, how it differs from similar concepts like fragility, the ethical arguments for discussing vulnerability in aged care ethics, and possible obligations following from recognising older adults’ vulnerability. We arrive at a more refined and nuanced systematisation of the different conceptualisations of older adults’ vulnerability as present in current bioethics literature as a result.

While vulnerability as a concept is a bit murky, some exceptions are represented by research ethics and, to a lesser extent, public health ethics. Within the former, the majority of scholars endorse the so-called ‘labelling approach’ [4, 13]. This approach labels a priori individuals or groups of individuals as being vulnerable according to the presence or absence of particular characteristics, namely, lack of capacity to consent to research, increased susceptibility to coercion or exploitation, and increased risk of harm. Criticizing such an approach as being too broad [77, 78] or too narrow [11], other scholars embrace ‘analytic approaches’ to understanding vulnerability [79]. Rather than identifying a priori populations, they aim to define sources and implications of vulnerability. Kipnis, for instance, proposes an account based on seven potential sources of vulnerability understood as ‘precariousness’ [80 p. 108], while Hurst suggests a wider range of remedies to ameliorate the vulnerability of research participants [3]. However, despite its promises, even this approach turned out to be less nuanced and context-based than originally envisaged.

In public health ethics, vulnerable populations are defined as ‘social groups who have an increased relative risk or susceptibility to adverse health outcomes’ [81 p. 70]. Although factors leading to such increased risk of adverse health outcome may vary, there is consensus in defining individuals as being vulnerable when they are incapable of safeguarding their own needs and interests adequately [82 p. 283]. Therefore, vulnerability is considered to be both a marker of social, political, and economic disadvantage correlated with poor health conditions, and/or as a feature of those who already have some forms of ill health, which increases their risk of further ill health.

Even within these domains, however, extensive debate failed to lead to the development of a comprehensive account of vulnerability [4]. Therefore, a satisfying and appropriate theorization is still missing. Reasons for this are plenty, spanning the contention that vulnerability should not be used as a substantive concept [5], to the acknowledgment of the difficulties in defining this concept [3], or even fixing its meaning in its broad-ranging connotations.

If applied to our specific context of investigation, no systematic and updated work on vulnerability, when referring to older adults, is present in the literature. Here, we present an analysis of relevant publications from 1984 to 2020 appearing in the philosophical, bioethical, and medical anthropology literature. The main findings are highlighted and discussed in depth next, with the ultimate aim of gaining a better understanding of how to properly deal with vulnerable older adults.

### Basic human vulnerability versus situational vulnerability: general considerations

The first main finding from our analysis is that the way vulnerability is conceptualised in the included publications is in line with the way the same concept is discussed in the current philosophical and bioethics literature. In the 38 publications we analysed, the authors tend to support one of two views of vulnerability. First, vulnerability is an inherent trait of human nature, an ontological condition of our own humanity, belonging to both embodiment – in turn related to propensity to disease and sickness, to impairment and disability, but also to death [7 p. 29] – and sociality and interdependence [9]. This is also referred to as basic human vulnerability. Second, vulnerability is the consequence of contingent unfair social, political, economic conditions; this also is referred to as situational vulnerability. While the first view of vulnerability has appeared in the literature under the guises of persistent [83], philosophical [1], anthropological [2], universal [9], or existential [39, 40] vulnerability, the second view has been mainly referred to as variable [83], political [1, 2], contingent [11, 39, 40], or extrinsic [11] vulnerability. Despite differences in their nomenclature, their content largely overlaps. Only in one publication did the author refer to both kinds of vulnerability [51], recognising that basic human and situational vulnerability are not necessarily mutually exclusive [51, 55, 84, 85]. His view of vulnerability is that the two may actually coexist, with one encompassing a more complex and multi-layered understanding of vulnerability (see below). These two views of vulnerability are mainly supported by different philosophical and bioethical approaches. Whereas the basic human vulnerability perspective is mainly advanced by authors leaning towards continental philosophy—in particular phenomenology and existentialism, and by advocates of ethics of care—the situational vulnerability perspective is advanced by authors advocating analytic philosophy and social science approaches.

In the context of aged care, supporters of basic human vulnerability tend to conceive of aging as a condition of increased basic human vulnerability, while supporters of situational vulnerability claim that aging may be conceived of as a condition of increased situational vulnerability. Despite their differences, both accounts share the idea that older adults already are, or may become, more vulnerable, because some of the conditions that determine vulnerability itself are increasing. For the basic human vulnerability view, these conditions include ever-increasing human finitude with age (experiencing human mortality more vividly). For situational vulnerability, these conditions include contingent extrinsic circumstances. Few exceptions exist, since the authors of four publications [36, 44, 51, 65] recognised the twofold nature of aging as a condition of both basic human vulnerability and situational vulnerability. Interestingly, our analysis showed that older adults’ vulnerability is never framed in terms of ‘pathogenic vulnerability’ [4], that is, vulnerabilities generated by misplaced or wrong answers to pre-existing vulnerabilities. However, if we focus on the ‘generators’ of pathogenic vulnerability’ as identified by Rogers and colleagues – namely, ‘morally dysfunctional interpersonal and social relationships characterized by disrespect, prejudice, or abuse, or by sociopolitical situations characterized by oppression, domination, repression, injustice, persecution, or political violence’ [4 p. 25] – then some interpretations of situational vulnerability may fall in this category. This occurs, for instance, with wrong answers to ‘relational/interpersonal vulnerability’ in case of dependent older adults. Older adults subjected to unnecessary hospitalisation or institutionalisation [56, 62, 69] – what we referred to as vulnerabilities related to ‘living situation’ – or illegitimately surveilled by others [58, 68] – what we referred to as vulnerabilities related to ‘conditions of control’ – may provide some examples of pathogenic vulnerability. Again, some forms of ‘moral vulnerability’ in its negative connotation may be considered as examples of pathogenic vulnerability: preventing older adults from receiving palliative care [57] as a consequence of stigmatisation goes precisely in this direction.

Another major finding of our analysis of the meaning of vulnerability in the context of aged care relates to dimensions of aged care vulnerability. We call these dimensions (i) physical; (ii) psychological; (iii) relational/interpersonal; (iv) moral; (v) sociocultural, political, and economic; and (vi) existential/spiritual vulnerability. By considering them as a whole, it becomes apparent that they map onto known defining features (and related vulnerabilities) of the human person. This observation gives weight to the view that vulnerability represents an inherent feature of humans, whereby being human and being vulnerable are largely coextensive notions. At the same time, however, the overlap between being human and being vulnerable is not a perfect match. The notion that the concept of vulnerability encompasses some fundamental traits of being human (e.g., experiencing finitude, being susceptible to physical and/or emotional wounds), does not mean that some other human traits realised in the future will also map onto the concept; they may not. It is recognised that this is true to the extent that being human is also defined, among others, by the capacity of being a rational agent, one endowed with moral agency, one looking for meaning, one open to transcendence, etc.

By defining vulnerability as a fundamental human trait, one may assume that vulnerability is a morally neutral notion. However, our analysis of the literature showed that vulnerability is mostly a value-laden concept, endowed with both positive and negative connotations. For example, vulnerability is clearly endowed with positive meaning when it is conceived of as a fundamental condition for human interconnectedness. This condition promotes genuine relations (relational/interpersonal vulnerability). Or when one’s vulnerability in late adulthood becomes an opportunity for authenticity, it may allow one to look at one’s life as acquiring new meanings (existential/spiritual vulnerability). On the other hand, this notion of vulnerability may take on a negative connotation insofar as it subjects older adults to infantilisation, depersonalisation, and objectification (moral vulnerability) but also to isolation, marginalisation, and stigmatisation (sociocultural vulnerability).

One of our research questions concerned the potential correlation between the acknowledgment of vulnerability and corresponding moral obligations. Accordingly, we asked whether vulnerability should be regarded as an independent moral concept, one that can generate moral obligations per se, or whether responses to vulnerability should necessarily appeal to other concepts or values (e.g., respect for persons, preventing harm). This also relates to other related questions, namely, whether moral obligations uniquely follow from the recognition of situational vulnerability, or are generated by basic human vulnerability as well; and to what, if any, these moral obligations amount to. This is a compelling and debated question in the philosophical and bioethical literature on vulnerability. The majority of scholars do claim that vulnerability is per se a source of moral obligations [86–88], though they variably interpret the content of such obligations, as well as the definition of who is responsible for fulfilling these obligations. Drawing from the consideration of vulnerability being related to relationships of dependency and interdependency which may expose human beings to harm and exploitation, Goodin, for instance, formulates the ‘Principle of protecting the vulnerable’ [86]. In his view, we have an obligation to act so as to prevent harms to, or protect the interest of, those who are especially vulnerable to our actions and behaviours [86]. More recently, Reader claims that vulnerabilities arise from needs, that we, as humans, all have [88–90]. However, in his view, not all needs are equally morally demanding, but only ‘vital needs’, the latter not being limited to bodily sustenance and protection, but defined broadly in relation to context-specific situations and personal identity [88]. On the other side of the spectrum, other scholars suggested that moral obligations cannot derive from vulnerability alone but from vulnerability-related factors. Hurst, for instance, argues that is ‘the increased likelihood of incurring additional harm or greater wrong’ [3 p. 195] which creates the obligation to protect those who are more than ordinarily vulnerable.

In our included papers, it is not only situational vulnerability [44, 45, 50, 51, 53, 56, 64, 65, 69, 71], but also basic human vulnerability [43, 44, 46, 53], that is considered a source of moral obligations. Regarding the content of moral obligations arising from vulnerability, most of the publications we analysed focused on and promoted aged care in broadly conceived terms [38–46, 48, 50, 52, 57, 59, 63, 68, 69, 72, 74, 75]. In particular, one’s attitude towards care, which is interpreted as both understanding the vulnerability of and protecting older adults, constitutes the most appropriate and fundamental response to vulnerability. This finding strengthens the idea that care is a defining practice of being human. Care and vulnerability are intrinsically linked as instances of humans. This does not mean, however, that vulnerability is ‘erased’ once it is recognised as such. Rather, it means that only after having recognised the existence of a universal basic human vulnerability and after having understood that some human beings are more vulnerable than others, a proper and targeted response may then be put in place. This is especially true for advocates of situational vulnerability.

### Older adults’ vulnerability as a multi-dimensional concept

When grappling with the concept of vulnerability in a concrete way, the authors end up integrating elements of both basic human vulnerability and situational vulnerability. This is the case even though almost all the authors claimed to endorse just one of the two accounts. It seems reasonable to suggest, then, that a binary understanding of the concept of vulnerability in aged care is inappropriate.

Our systematic review provides some evidence towards the understanding of vulnerability in aged care as preliminarily conceptualised by some scholars in contemporary bioethics. The current tendency is to characterise older adults’ vulnerability in terms of layers [84, 85, 91], or, as our analysis showed, in terms of dimensions of vulnerability, rather than populations that are a priori vulnerable. The a priori notion is still the prevailing tendency in research ethics [15–17], mainly for pragmatic and precautionary reasons.

This systematic review also revealed a perspective on vulnerability that holds that a stable (i.e., age-independent) dimension of vulnerability can be identified, one that pertains to all humans. This is reasonable since we all share the same (finite) human condition. However, this new understanding also allows for some dynamic dimensions of vulnerability. As dynamic dimensions are not necessary conditions, they may or may not be present. Situational conditions (e.g., socioeconomic, political, unfair conditions) and fundamental dimensions pertaining to human life (e.g., progressive bodily deterioration) determine this dynamism.

If a proper understanding of vulnerability is partly a matter of recognising that layers or dimensions of vulnerability exist, then vulnerability is not necessarily a black or white condition but may be a condition comprising various degrees of vulnerability. Moreover, such a view permits one to foresee conditions that render the same person, or category of persons, as situationally vulnerable in some instances but not in others. One may become more or less vulnerable when additional levels of vulnerability are acquired or lost during the course of life.

Finally, if vulnerability is a multidimensional and complex phenomenon, then the objections raised in bioethics literature to ‘monodimensional’ accounts of vulnerability, either situational or basic human, tend to collapse. Indeed, considering vulnerability only as a trait of human nature may have the implication of ‘naturalising’ vulnerability [91], considering it as something that ‘cannot be reduced or remedied’ [1 p. 397], that is, an unavoidable phenomenon related to human existence. On the other hand, interpreting vulnerability only as the consequence of situational or contingent situations, may present the danger of non-acknowledging some fundamental traits of the human condition, e.g., the precariousness deriving from being mortal, considering these traits as personal deficits, rather than essential human features. In other words, if vulnerability is both a defining characteristic of our own humanity and an additional condition affecting some people in specific contingent situations, then understanding vulnerability, taking care of vulnerable persons, and intervening through sociopolitical and economic measures all become strategies to be synergistically adopted to respond to vulnerability.

### Strengths and limitations

The main strength of this systematic review is the large number of publications that contributed to our analysis. Most of them were published in the last five years. Also, the analysed publications reported on studies conducted in countries across the globe. Therefore, we believe these characteristics strengthened our conclusions.

Although our study produced new insights into the meaning, foundations, and uses of vulnerability as an ethical concept in the literature on aged care, it had a few limitations. Some may question that the variety of expressions used (vulnerability, frailty, fragility) may have potentially created some confounding factors in results comparisons. However, we considered this variety to be acceptable as long as these notions were used as synonyms of vulnerability in the publications and were used within argument-based reasoning, rather than strictly as medical terms. Secondly, we considered only publications written in English. Moreover, this research was conducted before the Covid-19 pandemic, so any potential impact it might have had on the understanding and uses of the concept of vulnerability were precluded. Intuitively, it is reasonable to assume that older adults’ vulnerability was greatly impacted [92]; future ethical analyses will determine whether the Covid-19 pandemic prompts us to modify pre-pandemic conclusions. Investigations about older adults’ vulnerability before and after Covid-19 may be highly relevant for future lines of research, especially those drawn towards understanding how vulnerability as an ethical concept affects delivery of healthcare to older individuals.

## Supplementary Information

**Additional file 1**. Example of conceptual scheme.

## Data Availability

All data generated or analysed during this study are included in this published article in Table 4. Additionally, the full list of QUAGOL schema are not publicly available due to privacy concern but are available from the corresponding author on reasonable request.

## Abbreviations

PRISMA: Preferred reporting items for systematic reviews and meta-analyses
QUAGOL: Qualitative analysis guide of Leuven
