# Supplementary material for: The concept of vulnerability in aged care: a systematic review of argument-based ethics literature
Source: BMC Med Ethics. 2022 Aug 16;23:84. doi: 10.1186/s12910-022-00819-3 (PMC9379886; doi:10.1186/s12910-022-00819-3)
Supplement: Supplementary file 1 — Additional file 1. Example of conceptual scheme. [file 12910_2022_819_MOESM1_ESM.docx]

| 28 | O'Brolchain F. (2019) Robots and people with dementia: Unintended consequences and moral hazard. Nursing Ethics 26 4 962-972. |
| --- | --- |

| CONCEPTUAL SCHEME | |
| --- | --- |
| Focus | Vulnerability |
| Definition/Conceptualisation of Vulnerability | Vulnerability (together with affliction and dependence) constitutes one of the fundamental traits of human nature, (i.e. human/existential vulnerability); vulnerability is here interpreted as mutual dependence.  There is also another kind of vulnerability (although not explicitly defined in these terms), which is vulnerability as related to robots (in particular SARs). |
| Theoretical approach  (VIRTUE ETHICS) | Neo-Aristotelian Virtue Ethics |
| Supporting authors | Alasdair MacIntyre (in particular, his position as developed in the book “Dependent Rational Animals”) |
| Population | Older adults with dementia (PwD) |

CONCEPTUALISATION OF AGED CARE VULNERABILITY

One of contemporary frontiers of aged care is the introduction of SARs in the care of older people, including people with dementia (PwD). The author’s claim is twofold.

1. SARs tame some older adults’ vulnerabilities (e.g. “Using robot carers should at least reduce the likelihood of physical, psychological and sexual abuse”; “like many of the oldest old and people with intellectual disabilities, will need assistance with toileting, showering and dressing. The use of robots might protect privacy and dignity of PwD”; “the use of robots might have direct psychological benefits for patients and for family members” – p. 966) but, at the same time, they exacerbate new vulnerabilities (control; isolation; deception; unemployment – p. 966-968). 🡪 Vulnerability in relation to New Technologies.
2. Introducing SARs for PwD in the long run may deeply affect our society, leading towards a very robust detrimental change. Indeed, by having less personal interactions with PwD, we as individuals but also as society will become less cognisant of the centrality of vulnerability and dependence in human life 🡪 Vulnerability as a trait of Human Nature.

(THEOR)ETHICAL FRAMEWORK

- The underlying philosophical assumption is that recognising and experiencing this second kind of vulnerability is essential for the proper flourishing of human nature.
- The author draws on a neo-Aristotelian virtue ethics approach (based on MacIntyre “Dependent Rational Animals”, 1999) which posits the facts of vulnerability, affliction and dependence as central to the human condition.
- Within this account, the concept of virtue is indented in a twofold manner: i) virtu as what enables individuals from mere animal creatures to independent rational agents, but also ii) virtue as what enables individuals “to confront and respond to vulnerability and dependence in ourselves and others” (p. 968).
- Corresponding to these two meanings of virtue, MacIntyre thematised two kinds of virtues: i) the virtues of independent rational agency, central in contemporary moral philosophy (justice, temperateness, truthfulness, courage); ii) the virtues of acknowledged dependence (“the Lakota virtue, wancantognaka, a term that encompasses both generosity and justice, and misericordia, which translates as somewhere between pity and compassion”, for details see p. 969).

The intuition underlying acknowledged dependence is that only by experiencing other’s vulnerability (in this case by directly interacting with PwD) we may understand how vulnerable and dependent creatures we are. 🡪 Comprehensive Flourishing then requires a practical learning, which is built through social relationships, which allow us to develop both kinds of virtues.
